# ﻿An eastern Congolian endemic, or widespread but secretive? New data on the recently described *Afrixaluslacustris* (Anura, Hyperoliidae) from the Democratic Republic of the Congo

**DOI:** 10.3897/zookeys.1224.128761

**Published:** 2025-01-21

**Authors:** Tadeáš Nečas, Gabriel Badjedjea, Janis Czurda, Václav Gvoždík

**Affiliations:** 1 Institute of Vertebrate Biology of the Czech Academy of Sciences, Brno, Czech Republic Institute of Vertebrate Biology of the Czech Academy of Sciences Brno Czech Republic; 2 Biodiversity Monitoring Centre, Department of Ecology and Aquatic Biodiversity, University of Kisangani, Kisangani, Democratic Republic of the Congo University of Kisangani Kisangani Democratic Republic of the Congo; 3 Department of Botany and Zoology, Faculty of Science, Masaryk University, Brno, Czech Republic Masaryk University Brno Czech Republic; 4 Department of Zoology, National Museum of the Czech Republic, Prague, Czech Republic National Museum of the Czech Republic Prague Czech Republic

**Keywords:** Afrotropics, bioacoustics, Central Africa, distribution, frogs, leaf-folding frogs, phylogeography, reproduction, spiny reed frogs, tropical rainforests

## Abstract

The Great Lakes spiny reed frog (*Afrixaluslacustris*) was recently described from transitional (submontane) forests at mid-elevations of the Albertine Rift mountains in the eastern Congolian region. Previously, because of its similarity, it had been understood to represent eastern populations of the unrelated *A.laevis*, which is known mainly from Cameroon. Based on DNA barcoding, we document the westward extension of the known range of *A.lacustris* within lowland rainforests in the Northeastern and Central Congolian Lowland Forests. One sample was represented by a larva found in a clutch in a folded leaf, a typical oviposition type for most *Afrixalus* species, contrary to oviposition on an unfolded leaf surface in the similar *A.laevis* and closely related *A.dorsimaculatus* and *A.uluguruensis*. Comparison of the advertisement call of *A.lacustris* from Salonga National Park, Democratic Republic of the Congo, indicates similarity to its sister species from montane areas of the Albertine Rift, the ghost spiny reed frog (*A.phantasma*). Phylogeographic analysis suggests that *A.phantasma* and *A.lacustris* speciated allopatrically during the Early Pleistocene, with the former having refugia in montane forests and the latter in transitional and also lowland forests. The lowland populations of *A.lacustris* represent distinct evolutionary lineages, which diversified probably in isolated forest refugia during the Middle Pleistocene.

## ﻿Introduction

Although Central Africa has been in the viewfinder of researchers for more than a hundred years, one of its parts, the central Congo Basin located under the wide arc of the Congo River, is still a mostly ‘empty spot’ on a map regarding some groups of African fauna. One of these groups is amphibians, as only a single comprehensive study on the species diversity of amphibians of the Central Congolian Lowland Forests (sensu Burgess et al. 2004) has been published to date (Badjedjea et al. 2022). Spiny reed frogs or leaf-folding frogs (*Afrixalus* Laurent, 1944) are known from 37 species from around sub-Saharan Africa, and a majority of species are characterized by a remarkable oviposition type as they use skin secretions to ‘glue’ edges of leaves to form a ‘nest’ for their eggs (Channing and Rödel 2019). One of the few exceptions is *A.laevis*, which deposits its eggs on leaf surfaces without forming the ‘leaf nest’ (Amiet 2012). This species had been until recently understood as having a largely disjunct distribution in western (Cameroon, Gabon, Bioko Island) and eastern Central Africa (eastern Democratic Republic of the Congo, Rwanda, Uganda) (Schiøtz 1999), although discussions about potential species-level distinction of the eastern population occurred (e.g., Amiet 2009, 2012).

The first relatively comprehensive understanding of phylogenetic relationships of *Afrixalus* was introduced by Portik et al. (2019), who showed that *Afrixalus* (beside “*Afrixalus*” *enseticola* Largen, 1974) is formed by two main clades later marked by Conradie et al. (2020) as Clade A and Clade B. Among the species studied by Portik et al. (2019) was also “*Afrixaluslacustris*” from Uganda, at that time not yet formally described and thus introduced as a *nomen nudum*. Clade B of *Afrixalus* contains the type species of the genus *A.fornasini* (Bianconi, 1849) from Southeast Africa. Otherwise Clade B contains mostly species occurring in Central and West Africa, including *A.laevis* from Cameroon (Conradie et al. 2020; Nečas et al. 2022). Clade A consists of mostly minute species primarily from East and Southeast Africa. “*Afrixaluslacustris*” from Uganda was phylogenetically re-analyzed as A.cf.laevis and A.sp. aff.laevis (Conradie et al. 2020; Nečas et al. 2022), respectively, and was confirmed as belonging to Clade A in the proximity of *A.weidholzi* (Mertens, 1938) from West to northern Central Africa and *A.dorsimaculatus* (Ahl, 1930), *A.morerei* Dubois, 1986 and *A.uluguruensis* (Barbour & Loveridge, 1928) from the Eastern Arc Mountains of Tanzania (Channing and Rödel 2019). The “eastern *A.laevis*” (A.cf.laevis, A.sp. aff.laevis) was finally formally described by Greenbaum et al. (2022) as *A.lacustris* Greenbaum, Dehling, Kusamba & Portik, 2022, who also described its sister species *A.phantasma* Dehling, Greenbaum, Kusamba & Portik, 2022 from montane areas of the central Albertine Rift in the Democratic Republic of the Congo and Rwanda (> 1700 m a.s.l.), which was previously also confused with *A.laevis*. These authors also demonstrated that another Albertine Rift montane endemic, *A.orophilus* (Laurent, 1947), belongs to a closer phylogenetic relationship with *A.lacustris*.

The Great Lakes spiny reed frog (*Afrixaluslacustris*) is presently known from the eastern Democratic Republic of the Congo (DRC) and southern Uganda, but potential distribution in Rwanda and Burundi is anticipated (Greenbaum et al. 2022). It is known mainly from transitional forests (between montane and lowland forests, mid-elevations, < 1700 m) of the Albertine Rift and more rarely from open habitats, especially near Lake Tanganyika (Greenbaum et al. 2022). The authors of the species description also reported some records along the eastern edge of the lowland Congolian rainforest (e.g., Epulu, Ituri Province, DRC) and discussed that the species might be more geographically widespread in lowland rainforests than currently recognized. In particular, they discussed a geographically isolated record from Omaniundu in the eastern Central Congolian Lowland Forests of Sankuru Province, DRC, three males collected in 1959 (approx. 500 km from the nearest locality in eastern DRC; Laurent 1982; Greenbaum et al. 2022), which they assigned to *A.lacustris*. The authors, however, noted that due to its remote geographic origin and some morphological peculiarities, this population needs to be further investigated using molecular data. Thus, *A.lacustris* is biogeographically presently understood as an eastern Congolian species. Neither type of oviposition nor characteristics of advertisement call of *A.lacustris* have been described (Greenbaum et al. 2022).

In this study, we report a westward geographic range extension into the Congolian lowland rainforests, oviposition type, and advertisement call characteristics of this recently described, putative eastern Congolian endemic, *Afrixaluslacustris*.

## ﻿Material and methods

### ﻿Sampling

**Figure 1.**
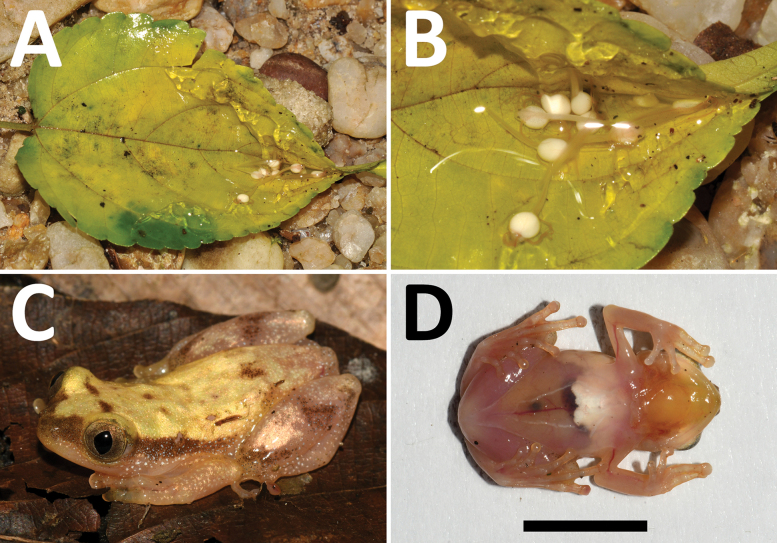
*Afrixaluslacustris***A, B** clutch with developing larvae, DNA barcoded (IVB-H-CD14-034), found in a folded leaf near Bafwabianga village, Tshopo Province, DRC. The leaf was picked, opened and photographed on the ground **C, D** adult male (IVB-H-CD23-0847) from near Isandja-Bomongili village, Salonga National Park, Tshuapa Province, DRC, in day-time coloration from dorsolateral and ventral view, the black bar corresponds to 10 mm.

We obtained two genetic samples of “Afrixaluscf.laevis” during our fieldwork in the Congolian lowland rainforests in DRC in 2014 and 2023. Several individuals in a very early larval developmental stage were collected from a gelatinous mass surrounding the egg clutch on a leaf and stored in 96% ethanol (IVB-H-CD14-034; IVB-H: herpetological collection in Studenec, Institute of Vertebrate Biology of the Czech Academy of Sciences, Brno, Czech Republic). The larvae were collected in the Dalangba Forest near Lindi River, near Bafwabianga village, Tshopo Province, northeastern DRC (1.1543°N, 26.8082°E, 510 m a.s.l.) on June 16, 2014 (Fig. 1A, B). No other sightings of “Afrixaluscf.laevis” were recorded during the extensive fieldwork of our team in the lowland rainforests of DRC, until a single adult male (IVB-H-CD23-0847) was recently found calling atop a *Ficus* tree above a shallow, partially dried up swamp near Isandja-Bomongili village, Salonga National Park, Tshuapa Province, central DRC (2.0526°S, 21.3832°E, 455 m a.s.l.) on October 23, 2023 (Fig. 1C, D). The male, after recording its advertisement call, was collected, euthanized, and its muscle tissue sample was stored in 96% ethanol.

### ﻿DNA barcoding

Species identities of the two samples were verified via DNA barcoding (Vences et al. 2012). Total genomic DNA was extracted using GeneJet Genomic Purification Kit (Thermo Fisher Scientific, USA). Fragments of the 16S rRNA gene (hereinafter *16S*) of mitochondrial DNA were amplified using polymerase chain reaction and the 16SL1 (forward) and 16SH1 (reverse) primers for the IVB-H-CD14-034 sample (Palumbi et al. 1991; 539 bp), and 16Sc (forward) and 16Sd (reverse) primers for the IVB-H-CD23-0847 sample (Evans et al. 2003; 871 bp) [note: 16SH1 and 16Sd bind to the same region]. Obtained *16S* sequences were compared with publicly available data (GenBank) using the BLAST search tool (Altschul et al. 1990). The most similar data were downloaded, aligned with our new data, and uncorrected *p*-distances were calculated using MEGA v. 11.0.13 (Tamura et al. 2021) after the complete deletion of gaps and missing data (Dolinay et al. 2021), resulting in 446 homologous sites. The newly generated sequences were deposited in the GenBank online database (IVB-H-CD14-034: PQ351303; IVB-H-CD23-0847: PQ351304.

### ﻿Phylogenetic analysis

To construct the phylogenetic tree, we used the same methodological approach as Greenbaum et al. (2022), reflecting their population-level divergence dating analysis, supplemented with our new data (Table 1). The analysis was performed on a 902 bp-long alignment using BEAST 1.10.4 (Suchard et al. 2018), GTR substitution model, coalescent tree prior, constant size growth prior, and a strict molecular clock set to 0.02 substitution/site per million years (Greenbaum et al. 2022). The analysis was run in triplicates for 10 million generations each, with sampling every 1000^th^ generation. The first 10% were discarded as burn-in, after convergence and effective sample size values were inspected using Tracer v. 1.7.2 (Rambaut et al. 2018), and a maximum clade credibility tree with median heights was created from the remaining post-burn-in 27,000 combined trees using LogCombiner 1.10.4 and TreeAnnotator 1.10.4 (Suchard et al. 2018).

**Table 1.** Origin of the *16S* sequences used in the dating analysis. Holotypes in bold. Abbreviations, collections: IVB-H (Institute of Vertebrate Biology of the Czech Academy of Sciences, Brno, herpetological collection Studenec, Czech Republic), UTEP (University of Texas at El Paso Biodiversity Collections, USA), CSB:Herp (Biodiversity Monitoring Center at University of Kisangani, herpetological collection, DRC), CAS (California Academy of Sciences, San Francisco, USA), ZFMK (Zoologisches Forschungsmuseum Alexander Koenig, Bonn, Germany); haplogroups: L (lowland), AR (Albertine Rift), N (north), S (south), C (central), NE (northeast); subgroups (a, b) of the AR-N haplogroup in parentheses.

*Afrixalus*	Collection No.	Locality	Haplogroup	*16S* GenBank	Reference
*A.lacustris*	IVB-H-CD14-034	DRC: Dalangba Forest, near Bafwabianga village	L-NE	PQ351303	This study
*A.lacustris*	IVB-H-CD23-0847	DRC: Isandja-Bomongili, Salonga National Park	L-C	PQ351304	This study
*A.lacustris*	**UTEP 20805**	DRC: Kalundu	AR-S	ON705200	Greenbaum et al. (2022)
*A.lacustris*	UTEP 20809	DRC: Baraka, Lake Tanganyika	AR-S	ON705201	Greenbaum et al. (2022)
*A.lacustris*	UTEP 22422	DRC: Baraka, Lake Tanganyika	AR-S	ON705204	Greenbaum et al. (2022)
*A.lacustris*	UTEP 22424	DRC: Itombwe Plateau, Mbandakila	AR-S	ON705217	Greenbaum et al. (2022)
*A.lacustris*	UTEP 22423	DRC: Kahuzi-Biega, Nanwa	AR-C1	PQ351598 ^†^	Greenbaum et al. (2022)
*A.lacustris*	UTEP 20810	DRC: Irangi	AR-C2	ON705199	Greenbaum et al. (2022)
*A.lacustris*	UTEP 22417	DRC: Toyokana	AR-N (a)	ON705198	Greenbaum et al. (2022)
*A.lacustris*	CSB:Herp:EPLU395	DRC: Epulu	AR-N (a)	ON705216	Greenbaum et al. (2022)
*A.lacustris*	UTEP 22416	Uganda: Bwindi, Buhoma	AR-N (a)	ON705206	Greenbaum et al. (2022)
*A.lacustris*	CAS 202036	Uganda: Bwindi, 2 km S of Bizenga River (by Buhoma rd.)	AR-N (a)	ON705208	Greenbaum et al. (2022)
*A.lacustris*	CAS 256035	Uganda: Bwindi, rd. N of Ruhija	AR-N (a)	ON705205	Greenbaum et al. (2022)
*A.lacustris*	DFH 1102*	Uganda: Kibale Forest, Ngogo Research Center	AR-N (b)	ON705203	Greenbaum et al. (2022)
*A.lacustris*	DFH 1103*	Uganda: Kibale Forest, Ngogo Research Center	AR-N (a)	ON705202	Greenbaum et al. (2022)
*A.lacustris*	CAS 256128	Uganda: Mabira Forest	AR-N (b)	ON705209	Greenbaum et al. (2022)
*A.lacustris*	CAS 256129	Uganda: Mabira Forest	AR-N (b)	ON705210	Greenbaum et al. (2022)
*A.lacustris*	CAS 256130	Uganda: Mabira Forest	AR-N (a)	MK509679	Portik et al. (2019)
*A.lacustris*	CAS 256131	Uganda: Mabira Forest	AR-N (b)	ON705207	Greenbaum et al. (2022)
*A.phantasma*	**ZFMK 103454**	Rwanda: Gishwati Forest	—	ON705212	Greenbaum et al. (2022)
*A.phantasma*	ZFMK 103455	Rwanda: Gishwati Forest	—	ON705211	Greenbaum et al. (2022)
*A.phantasma*	UTEP 20802	DRC: Kahuzi-Biega, ca. 4 km NW of Lwiro	—	ON705215	Greenbaum et al. (2022)
*A.phantasma*	UTEP 20803	DRC: Kahuzi-Biega, Mugaba	—	ON705214	Greenbaum et al. (2022)
*A.phantasma*	UTEP 20791	DRC: Nyakasanza Swamp near Tshibati	—	ON705213	Greenbaum et al. (2022)

^†^Submitted by T. Nečas, on behalf of Greenbaum et al. (2022). *Field No. (Daniel F. Hughes), photos and tissues only (Greenbaum et al. 2022).

### ﻿Acoustic recording and analysis

The advertisement call of the male (IVB-H-CD23-0847; snout-vent length, SVL = 21 mm) was recorded on a hand-held recorder Zoom H5 using a shotgun microphone Zoom SGH-6. The recording was obtained at 20:15 at 23.7 °C ambient temperature from a distance of 2–3 meters. The analysis of the recording was performed in SoundRuler v. 0.9.6 (Gridi-Papp 2007) and Raven Lite v. 2.0.5 (The Cornell Lab of Ornithology, Ithaca). The terminology of acoustic parameters is following Köhler et al. (2017). The recording was deposited in the FonoZoo online database with the reference number 14858 (https://www.fonozoo.com).

## ﻿Results and discussion

### ﻿Distribution and phylogeography

Due to the existence of the isolated record from Sankuru, some distribution maps of “*A.laevis*” sensu lato have shown the range as continuous from Cameroon, across the Congo, to southwestern Uganda (IUCN SSC Amphibian Specialist Group 2013; Channing and Rödel 2019). However, in reality, the range was always known as composed of two disjunct areas in the west (Cameroon, Gabon; *A.laevis* sensu stricto) and east (DRC, Rwanda, Uganda; *A.lacustris*, *A.phantasma*), with the Sankuru record located in between but closer to the eastern area (Schiøtz 1999). Greenbaum et al. (2022) assigned the Sankuru specimens to *A.lacustris*, but with a note that this population requires additional scrutiny with molecular data.

**Figure 2.**
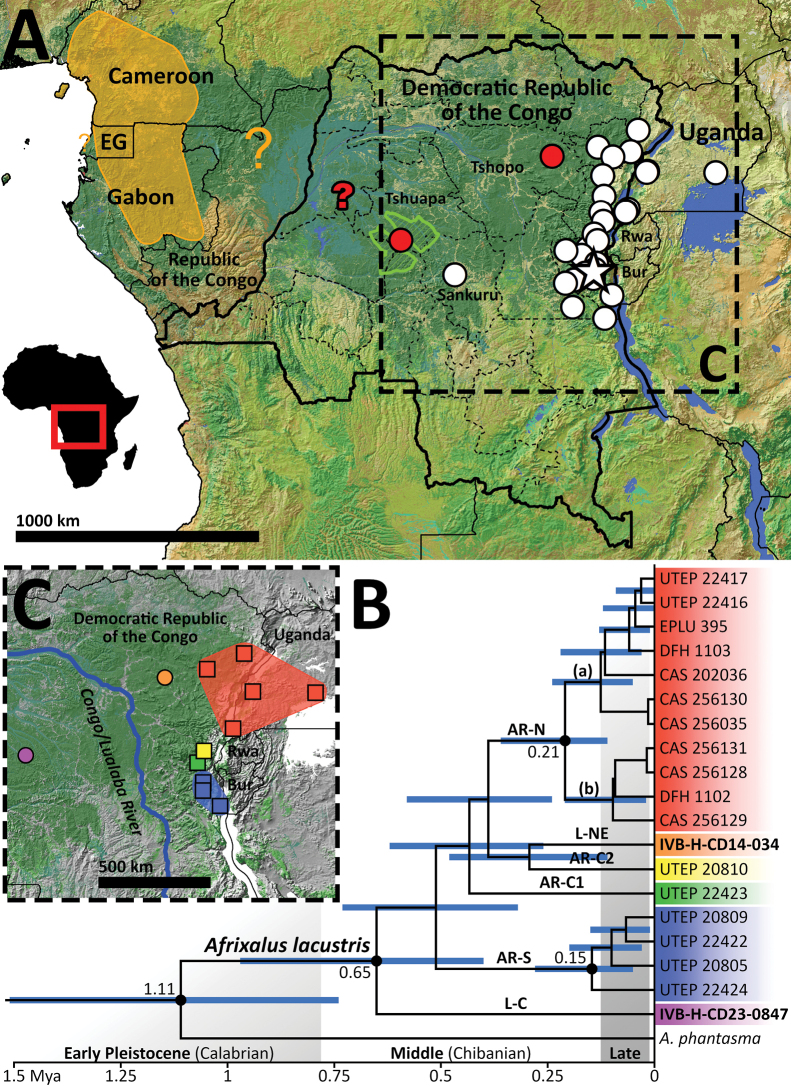
**A** map of all known distribution sites of *Afrixaluslacustris*. Red symbols mark new localities presented in this study; the red question mark denotes Boteka – a site in need of verification, where “*A.laevis*” was collected. Green polygons mark Salonga National Park. White symbols denote previously known localities of *A.lacustris* summarized by Greenbaum et al. (2022); the white star corresponds to the type locality. Orange polygons show the known distribution range of *A.laevis*, questionable areas are marked with orange question marks. EG = Equatorial Guinea, Rwa = Rwanda, Bur = Burundi **B** phylogenetic tree and **C** phylogeographic map of *A.lacustris* based on Greenbaum et al. (2022) with the addition of our new data (in bold). Black dots indicate highly supported nodes, numbers at nodes denote estimated divergence time (Mya), and blue bars denote 95% HPD intervals. A single representative of *A.phantasma* is shown as an outgroup. Haplogroups are distinguished by different colors and abbreviations placed on the branches (see Distribution and phylogeography, and Table 1). The maps were created in ArcGIS v. 10.8.1 (Esri Inc., https://www.esri.com), with land cover visualized by implementing results of the GlobCover project (Arino et al. 2012), and country and provincial boundaries and shaded relief background downloaded from https://naturalearthdata.com.

In this study, we present two new records of *A.lacustris* substantially extending its distribution in the Congo Basin westward into lowland rainforests, as the BLAST comparisons of *16S* retrieved *A.lacustris* as the most similar species for both our samples of “Afrixaluscf.laevis” (Fig. 2A, B). The IVB-H-CD14-034 sample was found to differ from the previously published *A.lacustris 16S* data by 1.5% uncorrected *p*-distance, the IVB-H-CD23-0847 sample differed by 1.9%, and the two samples differ from each other by 2.0%, which do not reach the 3% threshold suggested for identifying a potential candidate species in anurans (Vieites et al. 2009). The dated phylogenetic reconstruction performed on *16S* (Fig. 2B) shows a very similar topology to that of Greenbaum et al. (2022) although with slightly younger age estimates. The split between *A.lacustris* and its sister species *A.phantasma* is estimated to have occurred 1.11 million years ago, Mya (0.74–1.51 Mya, 95% highest posterior densities, HPD) during the Early Pleistocene (Calabrian). Six distinct mitochondrial lineages (haplogroups) with uncertain relationships are identified within *A.lacustris*, which originated during the Middle Pleistocene (Chibanian; Fig. 2B, C, Table 1). The first divergent lineage is represented by the single sample IVB-H-CD23-0847 from the Central Congolian Lowland Forests (L-C haplogroup). Its divergence is estimated to have occurred ~650 thousand years ago (hereinafter as kya; 400–970 kya) at the beginning of the Middle Pleistocene. The second diverging lineage consists of a population inhabiting the Albertine Rift in the south of the *A.lacustris* range (Itombwe Plateau, DRC, and its vicinity; AR-S haplogroup), from where the holotype originates. Diversification within the AR-S haplogroup is estimated to have occurred ~150 kya (50–280 kya), roughly corresponding with the beginning of the Late Pleistocene. The third lineage is a single sample from Kahuzi-Biega, DRC (UTEP 22423; AR-C1 haplogroup). The fourth and fifth lineages form a common clade (although with low support) and are represented by a sample from Irangi, DRC (near Kahuzi-Biega; UTEP 20810, AR-C2 haplogroup), and the sample IVB-H-CD14-034 from the lowland Dalangba Forest in northeastern Tshopo Province, DRC (L-NE haplogroup), respectively. The sixth mitochondrial lineage consists of samples from southwestern Uganda and adjacent DRC (haplogroup AR-N). Diversification within the AR-N haplogroup is estimated to have occurred ~210 kya (110–360 kya), at the end of the Middle Pleistocene. Two subgroups (a, b), with their diversifications corresponding with the beginning of the Late Pleistocene, are detectable in the AR-N haplogroup, but they have only weak supports (Fig. 2B, Table 1). The two subgroups have partially sympatric distribution in Uganda, and one subgroup (AR-N (b)) has been found only in Uganda, suggesting that a forest refugium was probably located in this area during the Late Pleistocene. The diversification into the six main mitochondrial lineages (haplogroups) of *A.lacustris* can probably be attributed to Middle Pleistocene climatic fluctuations and their impact on the suitable forest environment, which has undergone repeated fragmentations (Maley 1996; Zachos et al. 2001). However, the lack of statistical support for the relationships among the six identified mitochondrial lineages prevents further discussion on the historical biogeography of this species.

The range extensions lie in two areas (Fig. 2A). The first site is located in the north of the known *A.lacustris* range in Tshopo Province, approximately 200 km westward from the nearest locality (Epulu, Ituri Province; Greenbaum et al. 2022). The second site is situated in the south of the *A.lacustris* range, near the northern bloc of Salonga National Park in Tshuapa Province, and lies approximately 260 km to the northwest of the isolated record from Omaniundu in Sankuru Province (Laurent 1982). The discussed record from Omaniundu (as “*A.laevis*” in Laurent 1982), with specimens exhibiting partly distinct morphology (Greenbaum et al. 2022), thus probably indeed represents *A.lacustris*. Our two new records suggest that *A.lacustris* is probably rather widespread in the Northeastern and Central Congolian Lowland Forests. However, the paucity of its findings in the field—we had failed to find this species during every year of fieldwork between 2015 to 2022—confirms that *A.lacustris* hides excellently in the foliage of shrubs in dense forests, which explains why this species has not been detected in many areas despite its wide geographic distribution (Laurent 1982; Greenbaum et al. 2022). *Afrixaluslaevis* sensu stricto is similarly difficult to find (Greenbaum et al. 2022) and its southeastern extent is not well known. Besides Bioko Island, it is with certainty confirmed from Cameroon and Gabon (Greenbaum et al. 2022 and citations therein), but possibly extending deeper into the Congolian rainforests (Frost 2024). Unpublished data from the “Museum” database of the Royal Museum for Central Africa, which remains to be properly examined, indicate that “*A.laevis*” was also collected in 1985 in Boteka, Équateur Province, DRC (approx. 0.40°S, 19.11°E, 320 m a.s.l.). Whether this record represents an even more westward range extension of *A.lacustris*, an eastward extension of *A.laevis*, or potentially a new species, must yet be investigated.

### ﻿Oviposition type

The earlier larval sample (Fig. 1A, B) provides an important view into the reproductive biology of *A.lacustris*, as it uncovers that this species folds leaves to make a nest for its egg clutch. This is in line with its phylogenetic position, which is deeply divergent from its morphological convergent, *A.laevis*, which deposits eggs on leaf surfaces without folding the leaf (e.g., Amiet 2012). However, it is not known with certainty, besides the sister species *A.phantasma*, which other species are the most closely related to *A.lacustris*, whether the East African montane species *A.dorsimaculatus*, *A.morerei* and *A.uluguruensis*, or West African to northern Central African *A.weidholzi*, or *A.orophilus* from the Albertine Rift (Portik et al. 2019; Greenbaum et al. 2022; Nečas et al. 2022). *Afrixalusdorsimaculatus* and *A.uluguruensis* do not enfold egg masses in leaves, similar to *A.laevis*; *A.weidholzi* glues leaf edges into a nest during oviposition, while it is probably not known in *A.morerei* and *A.orophilus* (Harper and Vonesh 2002; Harper et al. 2010; Channing and Rödel 2019; Dehling et al. 2023).

### ﻿Advertisement call

**Figure 3.**
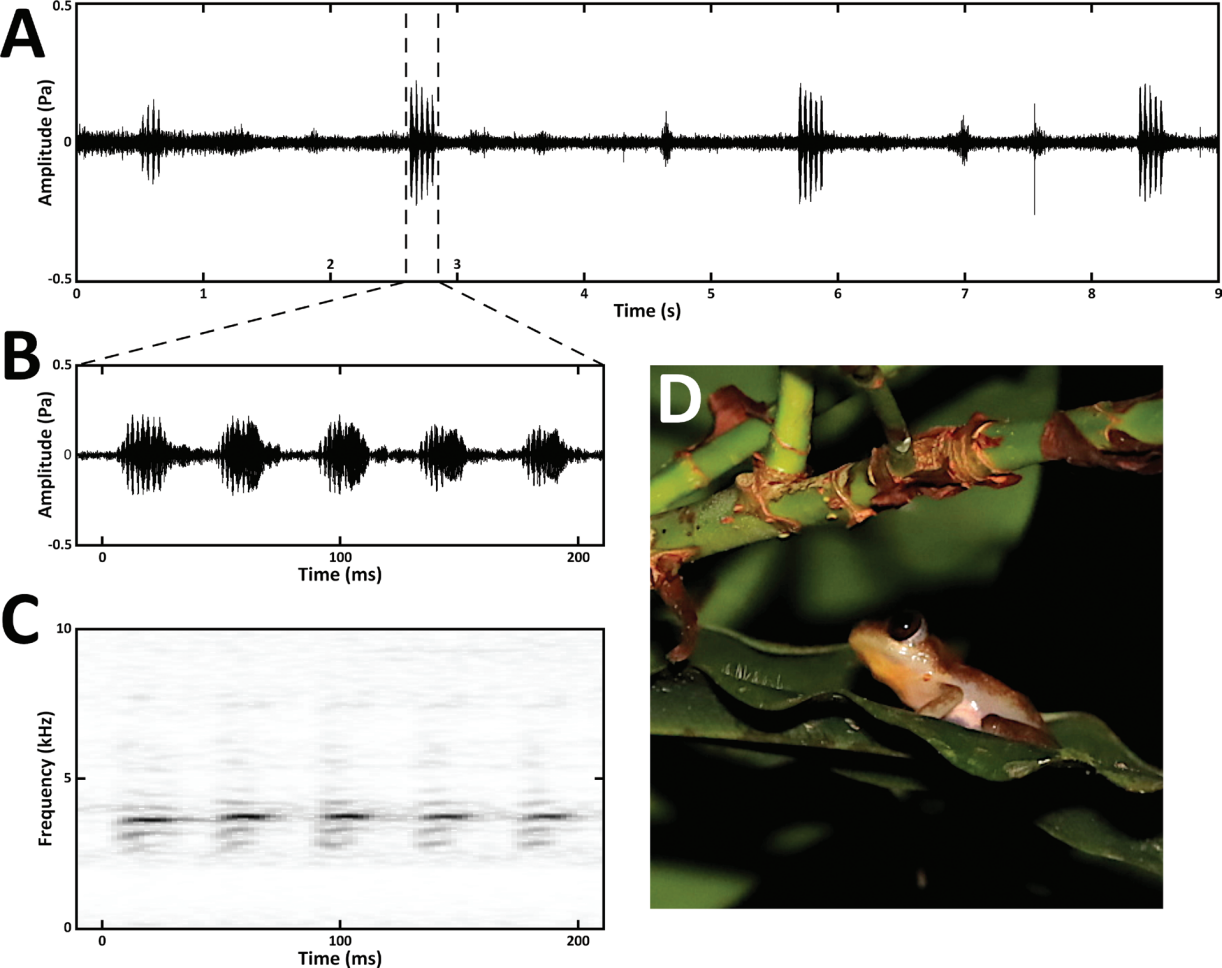
*Afrixaluslacustris* and its advertisement call **A** part of a call series (oscillogram, 9 s), with ambient frog chorus in the background **B** oscillogram of a five-note advertisement call **C** spectrogram of the respective call **D** the recorded male *A.lacustris* (IVB-H-CD23-0847) in nocturnal coloration in situ, perching on a leaf from where it was calling.

The second sample provides DNA-based identification of the calling male (SVL = 21 mm), which was initially found on a *Ficus* tree around 1.5–2 m above the ground, then disturbed, escaped and re-found on the top of the tree about 3 m above the swampy ground (Figs 1C, D, 3D). The advertisement call consists of four to five pulsed notes (4.8 mean for 37 measured calls of the single found male; Fig. 3A–C). Each note consists of 10 or 11 pulses, but the pulsation towards the end of the note was often obscured in the waveforms. Call duration in *A.lacustris* averages at 210 ms (195–220 ms, only five-note calls measured, *N* = 31; 167 ms average for four-note calls, *N* = 6; 23.7 °C). In the sister species *A.phantasma*, Greenbaum et al. (2022) documented longer durations of five-note calls (they reported five to six, rarely four notes per call), however their recordings were made in montane habitats at substantially lower temperatures with a trend of shortening call duration with increasing temperature (from 572–620 ms at 10.9 °C to 388–397 ms at 16.2 °C, five-note calls only). If we consider this trend in *A.phantasma*, our recording of *A.lacustris* made at 23.7 °C approximately fits with the mean call duration of 210 ms to a theoretically expected value in *A.phantasma* at the same temperature. The mean dominant frequency 3720 kHz (3627–3795 kHz) of *A.lacustris* is similar to the dominant frequency of *A.phantasma*, as measured at the higher temperature (3660–3810 kHz, 16.2 °C). Greenbaum et al. (2022) also observed a similarly obscured pulsation in the rear part of notes in *A.phantasma*, discussed as probably caused by echo effects. However, as we found a similar veiling in *A.lacustris*, and a similar characteristic was found in most of the notes in *A.orophilus* (Dehling et al. 2023), it is possibly a normal characteristic of the advertisement call in these species. In general, the advertisement calls of *A.lacustris* and *A.phantasma* are very similar, which is not surprising in sister species of frogs with parapatric distribution (see e.g. Gvoždík et al. 2015).

When we compare the advertisement call of *A.lacustris* with *A.laevis* (Amiet and Goutte 2017), with which it was previously confused, the general characteristics of the calls are quite different. Thus, the morphological convergence is not mirrored in the phonetic parameters, which might be related to partially different habitats. For example, reproduction of *A.lacustris* may occur more frequently in stagnant waters, whereas that of *A.laevis* in streams. If the advertisement call of *A.lacustris* is compared with other potentially closely related species (*A.dorsimaculatus*, *A.morerei*, *A.orophilus*, *A.uluguruensis*, *A.weidholzi*; Portik et al. 2019; Greenbaum et al. 2022; Nečas et al. 2022), none of them displays a substantial similarity of their advertisement calls; perhaps only *A.weidholzi* has partially similar call characteristics (Schiøtz 1999; Amiet and Goutte 2017; Dehling et al. 2023).

### ﻿Ecology and natural history

**Figure 4.**
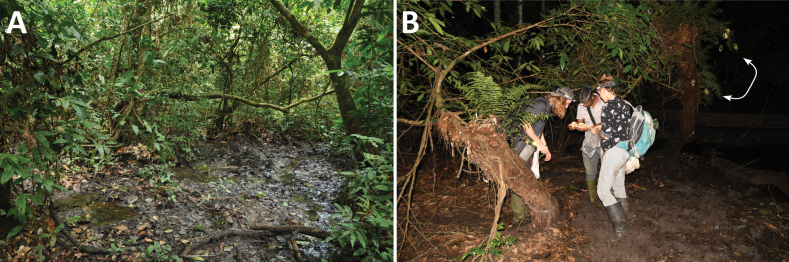
Habitats of *Afrixaluslacustris* in the Congolian lowland rainforests **A** Dalangba Forest, near Bafwabianga village, Tshopo Province, northeastern DRC**B** Isandja-Bomongili, Salonga National Park, Tshuapa Province, central DRC; junior authors after the finding of *A.lacustris*; note foam nests of Chiromantiscf.rufescens (white double arrow).

Despite our relatively intense fieldwork in the Congolian lowland rainforests during last 10 years (especially GB), we have found *A.lacustris* only twice, in two distant areas in Tshopo and Tshuapa provinces, in both cases based on single findings – one clutch with larvae and a single calling male. In the first case (Tshopo), the habitat was dense vegetation overhanging a drying muddy place near a sandy-bottomed stream in primary forest, where also small rainwater pools and puddles were present nearby, as well as a large river (Lindi; Fig. 4A). In the second case (Tshuapa), the habitat was a small drying swamp in a forest opening, located near a forest edge (local guides informed us that the swamp is much larger, potentially connected with flooded forest, after heavy rains; Fig. 4B). In both cases, habitats were in accordance with the *A.lacustris* habitat description of Greenbaum et al. (2022). Among sympatric amphibians, we found in Tshopo: Arthroleptidae: Arthroleptissp. aff.variabilis, *Arthroleptistuberosus* Andersson, 1905; Hyperoliidae: *Hyperoliusbolifambae* Mertens, 1938, *H.langi* Noble, 1924, *H.ocellatus* Günther, 1858; Pipidae: *Xenopuspygmaeus* Loumont, 1986, *X.ruwenzoriensis* Tymowska & Fischberg, 1973; Ptychadenidae: *Ptychadenachristyi* (Boulenger, 1919); Ranidae: Amniranacf.albolabris (Hallowell, 1856); and in Tshuapa: Arthroleptidae: Arthroleptissp. aff.variabilis, *Leptopelischristyi* (Boulenger, 1912); Hyperoliidae: Hyperoliuscf.kuligae Mertens, 1940 (eggs only), H.cf.veithi Schick, Kielgast, Rödder, Muchai, Burger & Lötters, 2010; Phrynobatrachidae: Phrynobatrachussp. aff.auritus; Pipidae: Hymenochiruscf.boettgeri (Tornier, 1896), *Xenopuspygmaeus*; Ptychadenidae: *Ptychadenaaequiplicata* (Werner, 1898); Ranidae: Amniranacf.albolabris; Rhacophoridae: Chiromantiscf.rufescens (Günther, 1869) (taxonomy and nomenclature follow Badjedjea et al. 2022). *Hyperolius* spp. at both sites and Chiromantiscf.rufescens were found with *A.lacustris* in syntopy on the same or nearby shrubs.

## ﻿Conclusions

*Afrixaluslacustris* is more widespread in lowland rainforests than previously thought. This suggests that the eastern Congolian fauna may be more widespread in the Central Congolian Lowland Forests. The lowland populations of *A.lacustris* are representatives of distinct evolutionary lineages that probably diversified in isolated forest refugia during the Middle Pleistocene. Along with the sister species *A.phantasma*, from which *A.lacustris* diverged during the Early Pleistocene, the Tanzanian montane species *A.dorsimaculatus* and *A.uluguruensis* are probably the most closely related species (Portik et al. 2019; Greenbaum et al. 2022) but are among the few exceptions in *Afrixalus* that lay eggs on the leaf surface and do not form nests. Here, we demonstrate that *A.lacustris* oviposits in folded leaves, as is common in most species of the genus. The advertisement call of *A.lacustris* has a similar structure to that of its parapatrically distributed sister species *A.phantasma*, whereas *A.dorsimaculatus* and *A.uluguruensis* have somewhat different calls. However, to better understand the evolution of reproductive behavior with respect to leaf-nest formation, as well as the evolution of advertisement calls, it is first necessary to better understand the species diversity of *Afrixalus* and the interspecific phylogenetic relationships, which are still not sufficiently known.

## ﻿Additional information

### Author ORCIDs

Tadeáš Nečas https://orcid.org/0000-0002-5060-8394

Gabriel Badjedjea https://orcid.org/0000-0003-4284-4327

Janis Czurda https://orcid.org/0009-0000-9151-3482

Václav Gvoždík https://orcid.org/0000-0002-4398-4076

### Data availability

All of the data that support the findings of this study are available in the main text and public databases.

## References

[B1] Altschul SF Gish W Miller W Myers EW Lipman DJ (1990) Basic local alignment search tool. Journal of Molecular Biology 215: 403–410. 10.1016/S0022-2836(05)80360-2 2231712

[B2] Amiet J-L (2009) Observations sur les *Afrixalus* du Cameroun (Amphibia, Anura, Hyperoliidae). Revue suisse de Zoologie, 116: 53–92. 10.5962/bhl.part.79490

[B3] Amiet J-L (2012) Les Rainettes du Cameroun (Amphibiens Anoures). J.-L. Amiet & La Nef des Livres, Nyons-Saint-Nazaire, 591 pp.

[B4] Amiet J-L Goutte S (2017) Chants d’Amphibiens du Cameroun. J.-L. Amiet & Editions Petit Génie, Nyons-Saint-Nazaire, 280 pp.

[B5] Arino O Ramos Perez JJ Kalogirou V Bontemps S Defourny P Van Bogaert E (2012) Global Land Cover Map for 2009 (GlobCover 2009). European Space Agency (ESA) & Université catholique de Louvain (UCL), PANGAEA. 10.1594/PANGAEA.787668

[B6] Badjedjea G Masudi FM Dudu Akaibe B Gvoždík V (2022) Amphibians of Kokolopori: an introduction to the amphibian fauna of the Central Congolian Lowland Forests, Democratic Republic of the Congo. Amphibian & Reptile Conservation 16: 35–70.

[B7] Burgess N Hales JDA Underwood E Dinerstein E Olson D Itoua I Schipper J Ricketts T Newman K (2004) Terrestrial Ecoregions of Africa and Madagascar. A Conservation Assessment. Island Press, Washington DC, 497 pp.

[B8] Channing A Rödel M-O (2019) Field Guide to the Frogs and Other Amphibians of Africa. Struik Nature, Cape Town, 408 pp.

[B9] Conradie W Keates C Lobón-Rovira J Vaz Pinto P Verburgt L Baptista NL Harvey J Júlio T (2020) New insights into the taxonomic status, distribution and natural history of De Witte’s Clicking Frog (*Kassinulawittei* Laurent, 1940). African Zoology 55: 311–322. 10.1080/15627020.2020.1821771

[B10] Dehling JM Mindje M Dumbo B Hinkel H Hinkel H Sinsch U (2023) Advertisement call and notes on the ecology of *Afrixalusorophilus* (Anura: Hyperoliidae) in Rwanda. Salamandra 59: 102–105.

[B11] Dolinay M Nečas T Zimkus BM Schmitz A Fokam EB Lemmon EM Lemmon AR Gvoždík V (2021) Gene flow in phylogenomics: Sequence capture resolves species limits and biogeography of Afromontane forest endemic frogs from the Cameroon Highlands. Molecular Phylogenetics and Evolution 163: 107258. 10.1016/j.ympev.2021.107258 34252546

[B12] Evans BJ Brown RM McGuire JA Supriatna J Andayani N Diesmos A Iskandar D Melnick DJ Cannatella DC (2003) Phylogenetics of Fanged Frogs: Testing Biogeographical Hypotheses at the Interface of the Asian and Australian Faunal Zones. Systematic Biology 52: 794–819. 10.1080/10635150390251063 14668118

[B13] Frost DR (2024) Amphibian Species of the World: an Online Reference. Version 6.2. [13^th^ May 2024]

[B14] Greenbaum E Portik DM Allen KE Vaughan ER Badjedjea G Barej MF Behangana M Conkey N Dumbo B Gonwouo LN Hirschfeld M Hughes DF Igunzi F Kusamba C Lukwago W Masudi FM Penner J Reyes JM Rödel M-O Roelke CE Romero S Dehling JM (2022) Systematics of the Central African Spiny Reed Frog *Afrixaluslaevis* (Anura: Hyperoliidae), with description of two new species from the Albertine Rift. Zootaxa 5174: 201–232. 10.11646/zootaxa.5174.3.1 36095401

[B15] Gridi-Papp M (2007) SoundRuler: acoustic analysis for research and teaching. https://www.soundruler.sourceforge.net (accessed 21 February 2024). Electronic database accessible at https://amphibiansoftheworld.amnh.org/index.php. American Museum of Natural History, New York, U. S. A. 10.5531/db.vz.0001

[B16] Gvoždík V Canestrelli D García-París M Moravec J Nascetti G Recuero E Teixeira J Kotlík P (2015) Speciation history and widespread introgression in the European short-call tree frogs (*Hylaarborea* sensu lato, *H.intermedia* and *H.sarda*). Molecular Phylogenetics and Evolution 83: 143–155. 10.1016/j.ympev.2014.11.012 25482363

[B17] Harper E Vonesh JR (2002) Field Guide to the Amphibians of the East Usambara Mountains. Preliminary Draft.

[B18] Harper EB Measey GJ Patrick DA Menegon M Vonesh JR (2010) Field Guide to the Amphibians of the Eastern Arc Mountains and Coastal Forests of Tanzania and Kenya. Camerapix Publishers International, Nairobi, 320 pp.

[B19] IUCN SSC Amphibian Specialist Group (2013) *Afrixaluslaevis*. The IUCN Red List of Threatened Species 2013: e.T56067A18370863. 10.2305/IUCN.UK.2013-2.RLTS.T56067A18370863.en [accessed 9 April 2024]

[B20] Köhler J Jansen M Rodríguez A Kok PJR Toledo LF Emmrich M Glaw F Haddad CFB Rödel M-O Vences M (2017) The use of bioacoustics in anuran taxonomy: theory, terminology, methods and recommendations for best practice. Zootaxa 4251: 1–124. 10.11646/zootaxa.4251.1.1 28609991

[B21] Laurent R (1982) Le genre *Afrixalus* Laurent (Hyperoliidae) en Afrique centrale. Annales du Musée Royal de l’Afrique Centrale. Tervuren, Belgique. Série in-8°, Sciences Zoologiques 235: 1–58.

[B22] Maley J (1996) The African rain forest – main characteristics of changes in vegetation and climate from the Upper Cretaceous to the Quaternary. Proceedings of the Royal Society of Edinburgh 104B: 31–73. 10.1017/S0269727000006114

[B23] Nečas T Kielgast J Nagy ZT Kusamba Chifundera Z Gvoždík V (2022) Systematic position of the Clicking Frog (*Kassinula* Laurent, 1940), the problem of chimeric sequences and the revised classification of the family Hyperoliidae. Molecular Phylogenetics and Evolution 174: 107514. 10.1016/j.ympev.2022.107514 35589055

[B24] Palumbi SR Martin A Romano S McMillan WO Stice L Grabowski G (1991) The Simple Fool’s Guide to PCR. University of Hawaii Press, Honolulu.

[B25] Portik DM Bell RC Blackburn DC Bauer AM Barratt CD Branch WR Burger M Channing A Colston TJ Conradie W Dehling JM Drewes RC Ernst R Greenbaum E Gvoždík V Harvey J Hillers A Hirschfeld M Jongsma GFM Kielgast J Kouete MT Lawson LP Leaché AD Loader SP Lötters S Van Der Meijden A Menegon M Müller S Nagy ZT Ofori-Boateng C Ohler A Papenfuss TJ Rößler D Sinsch U Rödel MO Veith M Vindum J Zassi-Boulou A-G McGuire JA (2019) Sexual dichromatism drives diversification within a major radiation of African amphibians. Systematic Biology 68: 859–875. 10.1093/sysbio/syz023 31140573 PMC6934645

[B26] Rambaut A Drummond AJ Xie D Baele G Suchard MA (2018) Posterior summarisation in Bayesian phylogenetics using Tracer 1.7. Systematic Biology 67: 901–904. 10.1093/sysbio/syy032 29718447 PMC6101584

[B27] Schiøtz A (1999) Treefrogs of Africa. Edition Chimaira, Frankfurt am Main, 350 pp.

[B28] Suchard MA Lemey P Baele G Ayres DL Drummond AJ Rambaut A (2018) Bayesian phylogenetic and phylodynamic data integration using BEAST 1.10. Virus Evolution 4: vey016. 10.1093/ve/vey016 PMC6007674 29942656

[B29] Tamura K Stecher G Kumar S (2021) MEGA11: Molecular Evolutionary Genetics Analysis version 11. Molecular Biology and Evolution 38: 3022–3027. 10.1093/molbev/msab120 33892491 PMC8233496

[B30] Vences M Nagy ZT Sonet G Verheyen E (2012) DNA barcoding amphibians and reptiles. In: Kress WJ Erickson DL (Eds) DNA Barcodes: Methods and Protocols.Methods in Molecular Biology, vol. 858, Humana Press, Totowa, 79–107. 10.1007/978-1-61779-591-6_5 22684953

[B31] Vieites D Wollenberg KC Andreone F Köhler J Glaw F Vences M (2009) Vast underestimation of Madagascar’s biodiversity evidenced by an integrative amphibian inventory. Proceedings of the National Academy of Sciences of the United States of America 106: 8267–8272. 10.1073/pnas.0810821106 19416818 PMC2688882

[B32] Zachos J Pagani H Sloan L Thomas E Billups K (2001) Trends, rhythms, and aberrations in global climate 65 Ma to present. Science (Washington) 292: 686–693. 10.1126/science.1059412 11326091

